# Toward interprofessional team training for surgeons and anesthesiologists using virtual reality

**DOI:** 10.1007/s11548-020-02276-y

**Published:** 2020-10-20

**Authors:** Vuthea Chheang, Virve Fischer, Holger Buggenhagen, Tobias Huber, Florentine Huettl, Werner Kneist, Bernhard Preim, Patrick Saalfeld, Christian Hansen

**Affiliations:** 1 Faculty of Computer Science and Research Campus STIMULATE, Otto-von-Guericke University Magdeburg, Magdeburg, Germany; 2 Department of Anesthesiology, University Medicine of the Johannes Gutenberg-University Mainz, Mainz, Germany; 3 Department of General, Visceral and Transplant Surgery, University Medicine of the Johannes Gutenberg-University Mainz, Mainz, Germany

**Keywords:** Medical training, Surgical simulation, Virtual reality, Mixed reality, Human–computer interaction

## Abstract

**Purpose:**

In this work, a virtual environment for interprofessional team training in laparoscopic surgery is proposed. Our objective is to provide a tool to train and improve intraoperative communication between anesthesiologists and surgeons during laparoscopic procedures.

**Methods:**

An anesthesia simulation software and laparoscopic simulation software are combined within a multi-user virtual reality (VR) environment. Furthermore, two medical training scenarios for communication training between anesthesiologists and surgeons are proposed and evaluated. Testing was conducted and social presence was measured. In addition, clinical feedback from experts was collected by following a think-aloud protocol and through structured interviews.

**Results:**

Our prototype is assessed as a reasonable basis for training and extensive clinical evaluation. Furthermore, the results of testing revealed a high degree of exhilaration and social presence of the involved physicians. Valuable insights were gained from the interviews and the think-aloud protocol with the experts of anesthesia and surgery that showed the feasibility of team training in VR, the usefulness of the system for medical training, and current limitations.

**Conclusion:**

The proposed VR prototype provides a new basis for interprofessional team training in surgery. It engages the training of problem-based communication during surgery and might open new directions for operating room training.

**Electronic supplementary material:**

The online version of this article (10.1007/s11548-020-02276-y) contains supplementary material, which is available to authorized users.

## Introduction

Virtual reality (VR) has great potential to support training tasks. The quality of anesthesia is crucial during surgery. An anesthesiologist along with the surgeons is responsible for a patient’s well-being by using medically measured vital signs during the procedures [1]. Therefore, anesthesia training aims to improve the anesthesiologist’s skills to allow surgical intervention without irreversibly harming the patient. Various VR simulations for surgical skill training have been proposed [2–4]. However, VR-based anesthesia training is underrepresented [5]. Complication-free narcosis during surgery is essential for a successful intervention. In particular, the quality of communication between anesthesiologists and surgeons during critical situations such as surgical complications is crucial [6].

The current standard for communication and team training of anesthesia is based on high-fidelity mannequins [7]. Training with mannequins involves an electrocardiogram (ECG) and vital signs, different levels of lung obstructions, and a simulation of the body’s reaction to a medication [8]. However, these training systems are not flexible concerning the aim of training [1, 9].

Collaborative VR can be used to improve the communication and teamwork during critical situations [7]. Nevertheless, current VR team training simulations for anesthesia provide inadequate interaction possibilities and limited training scenarios during laparoscopic procedures [10, 11]. The main contribution of our work is a prototype focused on training scenarios for VR-based interprofessional team training between surgeons, camera assistants, and anesthesiologists during laparoscopic procedures. We propose a layer as an interface to integrate a commercially standard anesthesia simulation software and a prototypical laparoscopic simulation developed in previous work [12] to tackle the problems discussed above. Furthermore, two scenarios, i.e., *undetected bleeding* (“Training scenario 1: undetected bleeding” section) and *insufficient muscle relaxant medication* (“Training scenario 2: insufficient muscle relaxant medication” section), are introduced. Clinical feedback is collected to assess the usefulness and limitations.

## Related work

In this section, we describe related work on VR-based anesthesia training and collaborative VR for interprofessional team training.

Prior work from Gaba et al. [13] introduced a full-scale anesthesia simulator called Comprehensive Anesthesia Simulation Environment. This simulation became a foundation for scenario-based simulations for skills training. Various VR-based anesthesia training simulators are used for clinical skills training such as diagnosing, monitoring, planning, and specific psychomotor tasks [14, 15]. Rare events or crisis management for training during laparoscopic procedures is performed [16]. Grottke et al. [17] developed a VR-based simulator for regional anesthesia training using multi-model representations in a large immersive virtual environment. Katz et al. [18] presented a serious game designed to teach anesthetic management of a standard orthotopic liver transplantation procedure. Furthermore, Shewaga et al. [19] developed a serious game for anesthesia-based crisis management training. However, their simulators are limited to the usage of single-person training that could make trainees believe that problems can be solved alone [20].

Collaborative VR emerged as an essential topic for research. However, there are a small number of collaborative VR applications for medicine, especially anesthesia training [21]. Paiva et al. [22] discussed the requirements to provide a collaborative VR-based simulator for surgical teamwork education. Most of the requirements, i.e., individual tasks, collaborative tasks, and simulated surgical procedure, are addressed in our prototype. Prasolova et al. [23] presented a virtual learning environment for interprofessional team communication and collaboration including the role of anesthesia. They found that students felt more engaged, motivated, and agreed that they had learned the value of clear communication. However, their results showed the limited interaction possibilities of using VR goggles. In our prototype, we used recent VR devices and developed various interaction possibilities, i.e., controlling the respirator and interactions to provide the medication. Cordar et al. [11] proposed a simulator that allows anesthesia residents to work with a mixed reality team that represented on large monitor screens. Furthermore, Brunges et al. [24] presented a similar mixed reality technology for interdisciplinary teams. Their results indicated that ineffective preoperative team member communication can result in serious patient harm. While Cordar and Bruges et al. focused on monitor-based solutions, our proposed prototype allows multi-user to connect and perform the training in the immersive environment.

As anesthesiologists are part of most surgical teams, their under-representation in medical VR scenarios seems critical. Additionally, there is not so much directly related work focused on interprofessional team training in medical VR, but that shows already the value of VR for team training. In this work, we introduce a VR prototype for interprofessional team training between surgeons and anesthesiologists.

## Material and methods

### System architecture

**Fig. 1:**
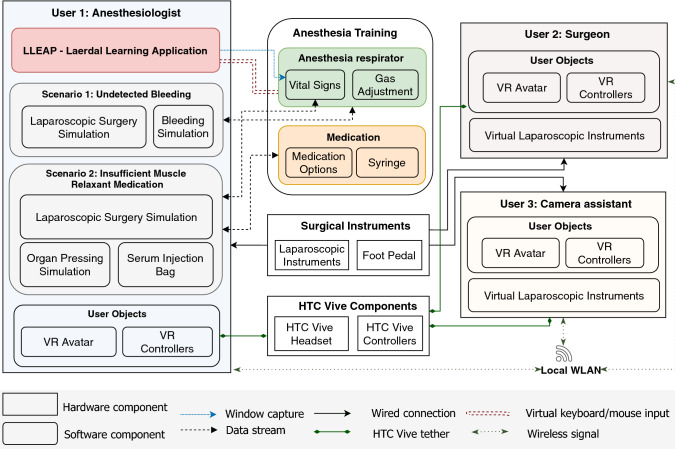
Architecture for multi-user VR simulation training in anesthesia and laparoscopic surgery

Figure 1 illustrates the architecture of our system. Three VR-ready computers are connected to a local wireless network. We use a client–server configuration, where a first computer acts as a server, and others are started as clients. However, the order of starting types (server or client) does not influence the user role. The same virtual objects are predefined for each user. When the user is connected, the related data and positions of objects will be synchronized to all users accordingly. The game engine Unity (version 2018.2.14) is used for development, and Unity networking (Unet) is applied for creating the multi-user environment. HTC Vive head-mounted displays are used to provide users with an immersive environment. Laparoscopic instruments (Simball joysticks, G-coder Systems, Sweden) and foot pedals are provided for the surgeon and camera assistant. When the user adjusts the anesthetic medication, an automated virtual mouse and keyboard input will change the anesthesia simulation software’s parameters to match the medication’s effects and scenarios.

### Anesthesia simulation software

**Fig. 2:**
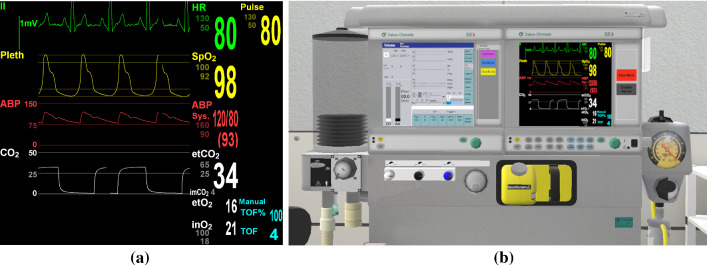
Vital signs monitoring of the system. The screen of anesthesia simulation software (**a**) is captured for the respirator’s right monitor (**b**)

The anesthesia simulation software LLEAP (Laerdal learning application, Laerdal Medical, Norway) is used as a component for vital sign monitoring in our multi-user VR for anesthesia training (see Fig. 2). LLEAP includes symptoms related to the developed scenarios such as vital sign changes for heart rate (pulse), arterial blood pressure (ABP), and *Train of Four* (TOF) as well as the measures of inhaled gases. Usually, LLEAP is used to control a high-fidelity mannequin. However, in this work, we integrated this software to simulate a virtual patient’s vital signs in VR. From the options provided in LLEAP, we choose *SimMan 3G* for vital sign monitoring. LLEAP provides a monitoring screen for vital signs in typical ranges and the specific curves, e.g., an electrocardiogram (see Fig. 2a). These vital signs can be altered and used as the monitoring screen. We set up a virtual respirator to monitor the vital signs in VR. The screen of LLEAP is captured and converted to a texture for the virtual screen of the respirator (see Fig. 2b).

**Fig. 3:**
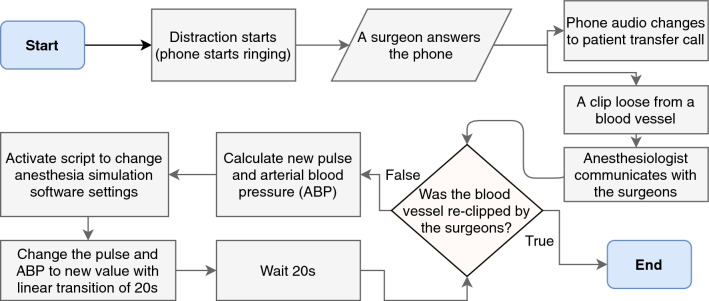
Flowchart for training scenario 1 (*undetected bleeding*)

### Training scenarios

To identify useful training scenarios and system requirements, we conducted an expert interview with our clinical partners, of which one is an experienced anesthesiologist, and two were laparoscopic surgeons. Simple tasks were implemented to provide a basic understanding (e.g., adjusting respiration gases, narcosis medication, and monitoring vital signs). Based on this interview, two scenarios were developed related to surgical complications: *undetected bleeding* and *insufficient muscle relaxant medication*. These scenarios should induce communication between the anesthesiologist and surgeons. For example, the anesthesiologist notes typical irregularities in the vital signs that may occur during bleeding and signals the surgeons that they should treat it. Moreover, a surgeon might detect a problem that the anesthesiologist is in charge of treating and tell her/him about it.

#### Training scenario 1: undetected bleeding

*Undetected bleeding* describes a situation in which a surgeon misses a bleeding during laparoscopic surgery. Hence, only the anesthesiologist can identify the problem by monitoring the change of vital signs. Then, the anesthesiologist is supposed to inform the surgeons about the issue, so the surgeon detects and stops the bleeding, e.g., by applying a clip onto the bleeding blood vessel.

**Table 1:** The implemented vital signs’ typical values and changes during specific situations

Situation	Pulse [bpm]	ABP systole [Hg/mm]	ABP diastole [Hg/mm]	TOF
Normal [25]	$\sim$ 80	$< 120$	$<80$	4
Narcosis	$\sim$ 59	$\sim$ 108	$\sim$ 64	$\le$ 2
Bleeding	Rises	Falls	Falls	No change
Low muscle relaxant	No change	No change	No change	Rises

*ABP* arterial blood pressure, *TOF* Train of Four

During laparoscopic surgery, some vascular structures need to be cut to resect a specimen, e.g., a liver tumor. However, a bleeding could lead to massive blood loss and endanger the patient. Furthermore, the surgeons’ field of view during the laparoscopy can be limited due to the bleeding. Thus, placing clips on blood vessels is a standard task during the surgery to avoid bleeding.

In some cases, these clips can loosen themselves and fall off. In the complicated situation of surgery, the surgeons may be distracted, e.g., by a phone call [26], and miss the resulting bleeding. Therefore, only the anesthesiologist can help by checking on the monitoring screen and informing the surgeons. Figure 3 illustrates the workflow of the scenario.

When the surgeons answer the phone, one of the clips previously placed on the blood vessels will slip off. During the bleeding, the ABP will drop and the pulse will rise as shown in Table 1. The anesthesiologist monitors these values and suspects bleeding and then notifies the surgeons to find and stop the bleeding accordingly. If the blood vessel gets clipped again, the bleeding will stop, and the vital signs will be stabilized.

#### Training scenario 2: insufficient muscle relaxant medication

This scenario aims to engage the surgeons to detect low muscle relaxation and make them inform the anesthesiologist to refresh the medication. It revolves around the patient starting to *press* as their abdominal muscles regain their ability to contract. If a patient starts pressing, the surgeons are more likely to harm the patient unintentionally. They will notice the pressing and tell the anesthesiologist about it so that the anesthesiologist can administer a refreshment dose of the muscle relaxant.

Usually, the anesthesiologist monitors muscle relaxation by evaluating the TOF. This value can vary significantly because it is dependent on the placement of the electrodes. Thus, it is possible that the anesthesiologist will not realize the low muscle relaxation herself and need a hint from the surgeons.

**Fig. 4:**
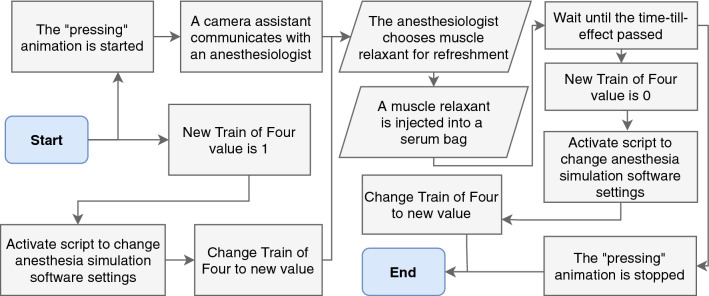
Flowchart for training scenario 2 (*insufficient muscle relaxant medication*)

The scenario starts with the animation for the *pressing* of the patient organs, parallel with the change in TOF parameters (see Fig. 4). With the injection of a new dose of muscle relaxant, the animations will stop, but only after the offset of the medication’s effect.

### Interactions for medication

**Fig. 5:**
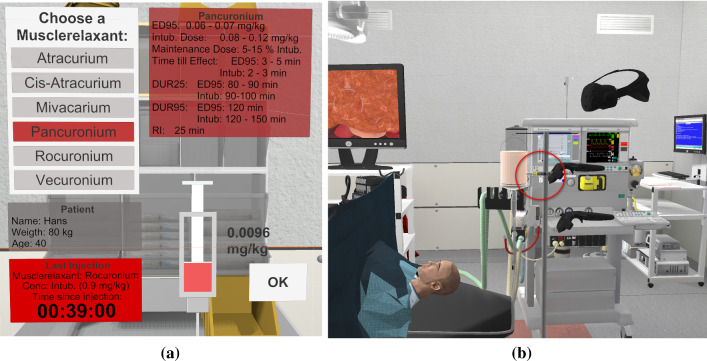
Interactions for refreshing the muscle relaxant medication: **a** muscle relaxant choice menu, **b** injection to a serum bag

In our simulation, the inhalation anesthetics can be operated and monitored at a virtual respirator. It is also possible to adjust the gas flow of oxygen, the carrier gas, and inhalation anesthetic with rotatable knobs (see Fig. 2b). Depending on the current minimal alveolar concentration (MAC) and inhalation anesthetic level, the ABP and pulse will change according to Eq. (1).

The vital medication for our system is the muscle relaxant because it is required as a countermeasure in scenario 2 (see Fig. 5). The muscle relaxant medication can be chosen from several options on an information panel with its type and dosage (see Fig. 5a). Afterward, the anesthesiologist is supposed to inject the medication with a virtual syringe to a serum bag beside the patient (see Fig. 5b).

### Change of vital signs

The vital signs of the anesthesia simulation software need to change realistically depending on the situation for the anesthesiologist to detect symptoms correctly. To replicate how vital signs change in reality, three different ways to affect the vital signs have been implemented (see the following subsections).

#### Changes of vital signs during narcosis

We use the dosage of the inhalation anesthetic to calculate the vital signs during narcosis. The effect of the dosage can be evaluated with minimal alveolar concentration (MAC) which is defined as the alveolar concentration of an anesthetic to prevent muscle movement. However, it cannot be predicted precisely how an individual would react to a specific amount of inhalation anesthetic. So the exact dosage relies on the anesthesiologist’s experience. Thus, a simple model was formed based on the following assumptions:

Each variation of the current inhalation anesthetic concentration $c_{IA}$ can be assigned to a specific value of pulse, systolic, and diastolic ABP. These will be called the target vital signs $x'$. They are defined by linear interpolation between the value for the average healthy pulse/blood pressure $x_\mathrm{norm}$ and pulse/blood pressure during narcosis $x_\mathrm{narc}$ as found in Table 1.

1 $$\begin{aligned} x' = x_\mathrm{norm} + \frac{c_{IA}}{MAC_{IA}} \cdot (x_\mathrm{narc} - x_\mathrm{norm}) \end{aligned}$$

By comparing the current vital sign *x* with the target vital sign $x'$, the absolute change can be calculated. However, the change of the vital signs will not take place instantly but over time. Therefore, a factor $0< m < 1$ can be chosen to calculate the absolute change of $\varDelta x$:

2 $$\begin{aligned} \varDelta x = (x' - x) \cdot m \end{aligned}$$

As a simple assumption, we set *m* to $\frac{1}{2}$. Finally, the next value of the vital sign $x'$ will be calculated by adding the change $\varDelta x$ to the current value of the vital sign *x*. The next vital sign $x'$ will be reached after 20 s following a linear transition.

**Fig. 6:**
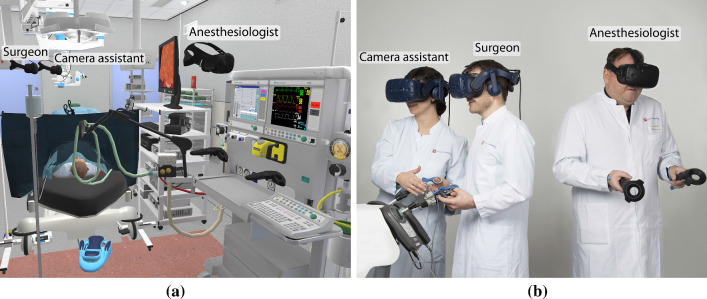
Overview of our interprofessional team training: **a** surgeon, camera assistant, and anesthesiologist virtually collaborate in VR, **b** users perform in the real world

#### Changes of vital signs during bleeding

The combined change of the pulse and ABP reliably indicates bleeding [27]. If the pulse exceeds the ABP, this is a safe sign for significant blood loss. However, the change of $\varDelta x$ should depend on the size of the vessel cut, the duration of the bleeding, and the bleeding speed. The change needs to be estimated as the bleeding factor *b*. It will symbolize the full effect of the parameters on the vital signs and results in a straightforward equation for the next vital signs $x'$.

3 $$\begin{aligned} x' = x + b \end{aligned}$$

The factor *b* is different for each vital sign as they need to change differently to indicate a bleeding:

$$\begin{aligned} b_\mathrm{pulse}= & {} 10 ~\mathrm {bpm} \\ b_\mathrm{ABP,sys}= & {} -8 ~\frac{\mathrm {mm}}{\mathrm {Hg}}\\ b_\mathrm{ABP,dia}= & {} -4 ~\frac{\mathrm {mm}}{\mathrm {Hg}} \end{aligned}$$

The changes are not abrupt and have a linear transition over 20 seconds from one state to the next.

#### Changes of vital signs during insufficient muscle relaxant medication

The value of TOF varies between zero and four. Usually, a TOF value of zero implies that the patient’s muscles are relaxed enough for a laparoscopy. Therefore, we chose value zero for regular narcosis. To display the muscle relaxant wearing off, the TOF value will change to 1.0 to indicate insufficient muscle relaxation. If the muscle relaxant is wearing off due to exceedance of the clinically effective duration (DUR25), the TOF value should change to 1.0 first, and the pressing will start a certain amount of time later.

## Evaluation

We performed a pilot study with an anesthesiologist with 27 years of working experience and two laparoscopic surgeons. The participants were asked to assess the simulation using the think-aloud method. The demonstration started with the basic functionalities needed for anesthesia. The anesthesiologist was asked to adjust the oxygen, carrier gas, and inhalation anesthetic in a way that he would think of as suitable for narcosis. The underlying processes were explained. Afterward, scenario 1 with the phone call and the vital sign stabilization was shown. Lastly, scenario 2 was demonstrated so that the pressing animation, as well as the options for choosing and injecting the muscle relaxant, was displayed. Figure 6 illustrates how the anesthesiologist and surgeons virtually joined and performed the training in VR. After the experts completed both scenarios, a detailed interview was held with the anesthesiologist for about one hour. The interview started with the general setup of the operating room (OR) used, the vital signs and monitoring, and the value of the different medications.

We also tested our system during the development with two surgeons and one medical student. This test’s objective was to measure the social presence with a cooperative social presence questionnaire [28], as this is essential for VR team training. This questionnaire includes scales of team identification, social action, motivation, and team value. The answers were rated with a five-point Likert scale from “not at all” (0) to “very much” (5). Participants were given equal weights to calculate the average scores. Moreover, feedback regarding technical aspects and usefulness from participants was collected for future adjustments of the prototype.

## Results

In the following, the expert’s feedback is described.

### Training scenarios

For training scenario 1, the patient’s condition needs to be identified as critical, so the ABP should fall below 80 (systolic) and 50 (diastolic) Hg/mm, and the pulse should rise above at least 100 bpm. The distraction was suggested to insert an animation or add another user to take and hold the virtual telephone. Surgeons usually step back from the patient, let go of the laparoscopic instruments, and fold their arms not to compromise sterility. This way, the distraction would ensure that the surgeons do not look at the laparoscopic screen, or the laparoscopic camera does not capture in the right position.

Aside from that, the pulse and ABP should increase slightly during scenario 2. The respiratory parameters usually show the patient’s spontaneous breathing and result in an alarm if there is something wrong. Thus, the respiration frequency will become unstable, and the respiration curve will show specific spikes. The expert was positive and evaluated both scenarios to engage communication between interprofessional teams.

### Interactions for medication

The opioids for analgesia were recommended to be included in the simulation because they are an essential part of the general anesthesia. The expert also stated that information screens should be added for different inhalation anesthetics similar to the muscle relaxant choice.

The options of the muscle relaxant help to show the different medications. Additionally, the expert remarked that the anesthesiologists do not usually fill the syringes during the surgery but instead have a broad choice of prepared syringes with different doses of muscle relaxant. However, it could be a basis for the trainees to learn the absolute *mg* values for the muscle relaxants.

**Table 2:** Overview of current limitations and possible solutions for future research, including clinical importance and technical viability

No	Current limitation	Possible solution	Clinical importance		T.v.
			Ane.	Sur.	
1	Movement of pressing animation is not sufficiently realistic	Animation of patient’s pressing should be slower and right directed	2	1	2
2	Synchronization of vital sign monitor to all users	Captured image should be compressed and decompressed over network	3	2	1
3	Alternation of vital signs during bleeding is not sufficiently realistic	Amount of change in the vital signs for pulse and ABP should be depending on the amount of blood loss	1	1	2
4	No opioid medication	Implement in the same way as muscle relaxant medication	1	3	3
5	No patient history information	Adding patient information panel including prior diagnose and treatments	1	2	3
6	Supervisor menu is needed for assessment during the session	Monitoring menu with possibilities to trigger the complication scenarios anytime during the simulation and documenting the information for evaluation	1	1	1
7	Collaboration in local network connection is unstable	Optimize network latency and data synchronization for remote collaboration	1	1	1
8	Only effective doses can be given for muscle relaxant	Enable underdosing/ overdosing of muscle relaxant	1	2	3
9	Missing haptic feedback from anesthesia objects (syringe, respirator, serum bag ...)	Using appropriate haptic device, e.g., data glove	2	3	1
10	Change team setup in the operating room according to surgical procedure, e.g., supine split-leg position (French position) ...	Enable pre-installed standard operating room settings	3	1	2

*Ane.* anesthesia, *Sur.* surgery, *T.v.* technical viability

Range of clinical importance rating: 1: high importance, 2: importance, 3: low importance. Range of technical viability: 1: difficult, 2: normal, 3: feasible

### Usefulness for medical training

The expert confirmed that the scenarios would encourage communication between the anesthesiologist and surgeons. The expert was asked how he would use our system as an instructor. He mentioned that he wants the students to have a clear goal for the simulation, which should be explained with the patient’s history before starting the simulation. So a patient history is needed and shown before starting the simulation. The instructor also needs some methods of supervision. Documentation of the simulation session is needed, so they can evaluate the students and give them feedback. An assessment to comment and grade the students should be included. The expert also liked the idea to induce a specific scenario any time during the simulation with an instructor’s control panel.

### Cooperative social presence

The results of the questionnaire were divided into four categories. First, *team identification* (*M* 4.13, *SD* 0.64) refers to the level of psychological attachment felt by a team member toward the others. We implemented voice chat and representation model in VR. Nonetheless, the prototype could be improved regarding the flexibility of team setup in the operating room which allows a user to identify the other user roles in the surgical procedures (see Table 2 No. 10.). Second, *social action* (*M* 4.08, *SD* 0.83) involves communication and contribution between interprofessional team members. All participants agreed that they had a mutual understanding. Third, *motivation* (*M* 4.06, *SD* 1.06) describes the action and responsibility to motivate the team. We found that the participants were positive about our proposed scenarios that induced the communication and responsibilities between interprofessional team. Finally, *team value* (*M* 4.44, *SD* 0.70) refers to the behavior, encouragement, and performance of the team during the training. The average score of team value was higher than other categories obtained in the questionnaire. This could demonstrate the value and benefits of interprofessional team training. Interestingly, two questions “My actions were determined by the objectives of the team” and “I felt my team shared a common overall aim” were rated with the highest score from all participants. The results show that social presence positively impacts on the training experience. Furthermore, the presence of other members in VR influences cognition and behavior of the trainees.

Apart from the questionnaire, the participants provided valuable insights which are relevant to assist defining the limitations and possible solutions in Table 2. There was no inconsistency between the student and experts because the feedback is mostly focused on technical aspects. Participants agreed that team training in VR could engage and motivate the learning procedures, particularly in a complex situation that requires efficient communication.

## Discussion and conclusion

Valuable insights were gained during the testing using the think-aloud protocol and the interview. The feedback from the expert anesthesiologist and surgeons was overall positive, and they appreciated the broad range of functionalities.

The essential features of the scenarios were implemented. The exhilaration experience is the key to increase the attractiveness and motivation in the immersive VR training. Moreover, a summary of the current technical limitations and possible solutions is shown in Table 2, with the ratings of clinical importance for anesthesia, surgery, and technical viability. The scores were determined by one anesthesiologist (H.B.) and two laparoscopic surgeons (T.H. and F.H.).

One of the possible challenges of our anesthesia training is that the system could give students an imprecise impression on how the vital signs change. While the change of vital signs is modeled based on typical values extracted from the literature, not every patient reacts in the same way toward medication or bleeding. Therefore, the patient’s history and medical records could be added for the training. Besides, the realism of the pressing animation, doses adjustment, opioid medication, haptic feedback, and flexible team setup in the OR could be improved for the effectiveness of training. Nonetheless, optimization of network latency for remote collaboration, the realism of vital signs during bleeding, and a supervisor interface for assessment are of high importance for both anesthesia and surgery.

Our prototype was evaluated with a small number of samples, and results are subjective. However, we intend to carry out a full clinical study in the near future. The potential objective measurements for future studies could be task completion time, error rate, and task performance [29, 30]. For instance, the anesthesiologist could be evaluated by the amount adjustment and type of medication, time from selection to applying the medication, and response time after the clip loss. The camera assistant could be evaluated by identifying aspects of camera navigation such as camera centering and steadiness of camera movement, and response time, while the depth of anesthesia is flattened [31]. The surgeon could be evaluated with respect to the time to apply the clips on bleeding vessels, the amount of blood loss, and the distance to risk structures or their damage [32].

The multi-user VR simulation is useful for medical training; however, the devices are still expensive, and development is time-consuming while the anesthesiologist’s tasks, such as monitoring vital signs, could be utilized in a simple 2D display. Nevertheless, our system can be used for training, refreshing the skills, and developing better teamwork in the future.

To sum up, a multi-user VR setup for interprofessional training in laparoscopic surgery is introduced. This system provides benefits for the training, such as real-time collaboration and communication, to enhance teamwork between the anesthesiologist and surgeons. We developed two scenarios to induce and engage the trainees to communicate during potential surgical complications. Insights gained from an interview with experts of anesthesia and surgery revealed the limitations and goals for future investigation. The proposed system opens new directions for medical training and provides a basis for future extensive clinical evaluation.

## Electronic supplementary material

Below is the link to the electronic supplementary material.

Supplementary material 1 (pdf 65 KB)

Supplementary material 2 (pdf 44 KB)

Supplementary material 3 (mp4 64566 KB)
