# Supplementary material for: Toward interprofessional team training for surgeons and anesthesiologists using virtual reality
Source: Int J Comput Assist Radiol Surg. 2020 Oct 20;15(12):2109–18. doi: 10.1007/s11548-020-02276-y (PMC7671979; doi:10.1007/s11548-020-02276-y)
Supplement: Supplementary file 1 — Supplementary material 1 (pdf 65 KB) [file 11548_2020_2276_MOESM1_ESM.pdf]

Table 1 The average rate on five-point Likert scale and related items from cooperative social presence questionnaire [1]

	Mean (SD)
**Team Identification**	**4.13 (0.64)**
Q1. I acted with my teammates in mind	3.67 (0.58)
Q2. I considered my teammates possible plans/thoughts	4.00 (1.00)
Q19. I felt a social connection to my teammates (camaraderie)	4.00 (0.00)
Q21. I felt like I was part of a team	4.33 (0.58)
Q23. I was aware of my team	4.67 (0.58)
**Social Action**	**4.08 (0.83)**
Q6. I felt my teammates were looking out for me	3.00 (1.00)
Q7. I felt I contributed to the team	4.67 (0.58)
Q8. I felt my actions made a difference to my teammates	4.67 (0.58)
Q11. The actions of my teammates affected my thoughts and actions	4.67 (0.58)
Q13. My team communicated well	3.33 (0.58)
Q14. The team had a mutual understanding	4.00 (0.00)
Q16. My teammates played a significant role in my experience of the simulation[1]	4.33 (0.58)
Q25. I felt the team helped me	4.00 (1.00)
**Motivation**	**4.06 (1.06)**
Q5. I did not want my team to think I had let them down	3.33 (1.53)
Q9. My actions were determined by the objectives of the team	5.00 (0.00)
Q12. Being part of a team motivated me	4.00 (0.00)
Q15. I put the performance of the team over my personal performance	4.00 (1.73)
Q17. I wanted my team to value me	3.67 (1.15)
Q18. I felt responsible for achieving the objectives of the team	4.33 (0.58)
**Team Value**	**4.44 (0.70)**
Q3. It was as much about the team as about my own game	4.00 (1.00)
Q4. I felt my team shared a common overall aim	5.00 (0.00)
Q10. I felt my team was committed to working together	4.67 (0.58)
Q20. I made an effort to work with my teammates	4.33 (0.58)
Q22. I felt my team shared common short term goals	4.67 (0.58)
Q24. My teammates were useful	4.00 (1.00)

References

1. Hudson M, Cairns P (2014) Measuring social presence in team-based digital games. Interacting with Presence: HCI and the Sense of Presence in Computer-Mediated Environments pp 83–101

[1] Revised item (Change the word game to simulation)
