# Supplementary material for: Toward interprofessional team training for surgeons and anesthesiologists using virtual reality
Source: Int J Comput Assist Radiol Surg. 2020 Oct 20;15(12):2109–18. doi: 10.1007/s11548-020-02276-y (PMC7671979; doi:10.1007/s11548-020-02276-y)
Supplement: Supplementary file 2 — Supplementary material 2 (pdf 44 KB) [file 11548_2020_2276_MOESM2_ESM.pdf]

Interview Questions

1 Topic: Realism

1.1 Training scenario 1: Undetected Bleeding

- Do the vital signs and animations used precisely depict the scenario "Undetected Bleeding"?
- What was missing, or how could the illustration be improved further?

1.2 Training scenario 2: Insufficient Muscle Relaxant Medication

- Do the vital signs used precisely illustrate the scenario "Insufficient Muscle Relaxant Medication"?
- Was the usage of muscle relaxant depicted well enough?
- What was missing, or how could the illustration be improved further?

1.3 Functionalities

- Please rate the realism of the possibilities and amount of change of the following vital signs and parameters:
  - Pulse/ Heart Frequency
  - Arterial Blood Pressure
  - Train of Four
  - O_2 flow
  - N_2O flow
  - Inhalation Anaesthetic
  - Muscle Relaxant
- Were there enough functionalities? What did you miss?

1.4 Interactions

- Were the following interaction possibilities intuitive or difficult to learn?
  - Teleportation
  - Controller-Interactions with objects
  - Laser-Interactions with menus
  - Laser-Interaction with objects
- In which aspect did you find the interactions challenging? If so, what changes would you suggest to improve interaction?

2 Topic: Communication

2.1 Training scenario 1: Undetected Bleeding

- Do you think the scenario "Undetected Bleeding" will encourage communication?
- What could be improved to encourage communication?

2.2 Training scenario 2: Insufficient Muscle Relaxant Medication

- Do you think the scenario "Insufficient Muscle Relaxant Medication" will encourage communication?
- What could be improved to encourage communication?

3 Topic: Teaching

- Can you imagine yourself using an application like this for teaching in the future?
- What should be included so you would utilize that?
- Which options of supervision would you like to have as a teacher/ instructor?
- Which options of control would you like to have as a teacher/ instructor?

- What were the positive features of the application?
- What were the negative features of the application?
- Do you have any recommendations to improve the application?
